# Serum 25-Hydroxyvitamin D and Depressive Symptoms in a 12-Year Korean Occupational Cohort: A Threshold Effect Confined to Severe Deficiency

**DOI:** 10.3390/nu18183012

**Published:** 2026-09-15

**Authors:** Si-Ho Kim, Seung-Hwan Kim, Chang-Ho Chae

**Affiliations:** 1 Division of Infectious Diseases, Samsung Changwon Hospital, School of Medicine, Sungkyunkwan University, Changwon 51353, Republic of Korea; siho.kim@samsung.com; 2 Department of Neurosurgery, Samsung Changwon Hospital, School of Medicine, Sungkyunkwan University, Changwon 51353, Republic of Korea; aajechtkskim@hanmail.net; 3 Department of Occupational and Environmental Medicine, Samsung Changwon Hospital, School of Medicine, Sungkyunkwan University, Changwon 51353, Republic of Korea

**Keywords:** vitamin D, 25-hydroxyvitamin D, depression, CES-D, sleep quality, occupational cohort, repeated measures, threshold effect

## Abstract

**Background/Objectives:** Low serum 25-hydroxyvitamin D [25(OH)D] has been proposed as a modifiable risk factor for depression, but the shape of the exposure–response relationship and its independence from sleep disturbance have not been characterised in a large occupational cohort with repeated measures. **Methods:** We analysed 246,751 health examinations of 62,989 Korean adults over 12 annual waves (2014–2025). Depressive symptoms were measured with the Center for Epidemiologic Studies Depression Scale (CES-D) and sleep quality with the Pittsburgh Sleep Quality Index (PSQI). Hierarchical linear and logistic regressions with cluster-robust standard errors (228,520 complete-case observations from 60,403 individuals) modelled serum 25-hydroxyvitamin D [25(OH)D], both continuously and categorically; restricted cubic splines tested non-linearity, and a random-intercept model estimated the intraclass correlation coefficient (ICC). **Results:** Mean 25(OH)D was 21.08 ± 8.71 ng/mL; 51.3% were deficient (<20 ng/mL) and 10.5% scored CES-D ≥ 16. The exposure–response relationship was non-linear (*p* = 0.001 for non-linearity). Relative to sufficiency (≥30 ng/mL), only severe deficiency (<10 ng/mL) was independently associated with depression (adjusted odds ratio [OR] 1.20, 95% confidence interval [CI] 1.09–1.32); deficiency (OR 1.05) and insufficiency (OR 1.02) were not. The per-1 ng/mL association was small (OR 0.996, 95% CI 0.993–0.999). PSQI score was the dominant predictor (OR 1.372 per point). The ICC was 0.743, and the within-person correlation (r = −0.008) was one-sixth of the between-person correlation (r = −0.046). **Conclusions:** The vitamin D–depression association is confined to severe deficiency rather than graded across the normal range, is small in absolute terms, and is substantially exceeded by that of sleep disturbance. Screening and repletion should target the severely deficient minority.

## 1. Introduction

Depression is a leading cause of disability worldwide, affecting more than one billion people when anxiety disorders are included and accounting for 125 million disability-adjusted life years in 2019 [1,2]. In the Republic of Korea, clinically significant depressive symptoms have become more common in successive national surveys [3], a trend attributed in part to occupational stress, reduced daylight exposure associated with urban indoor work, and disrupted sleep. Identifying genuinely modifiable determinants is therefore a public health priority [4].

Working populations are a particularly informative setting for this question. Predominantly indoor occupations limit ultraviolet-B exposure and are associated with a high prevalence of vitamin D deficiency, while long working hours, shift work, and occupational stress make sleep disturbances common. The two exposures therefore co-occur precisely in the group in which they are most often left unmeasured together. A large occupational screening cohort with concurrent measurement of 25(OH)D, depressive symptoms, and sleep quality offers the opportunity to estimate the association of each while adjusting for the other and to separate stable between-person differences from within-person change over time—the central aims of the present study.

Vitamin D, a fat-soluble secosteroid synthesised upon ultraviolet-B exposure, has plausible neurobiological effects beyond calcium homeostasis. Vitamin D receptors are expressed in the prefrontal cortex, hippocampus and cingulate gyrus [5]; 25(OH)D facilitates serotonin synthesis through upregulation of tryptophan hydroxylase-2 [6]; and vitamin D promotes hippocampal neuroplasticity, partly through brain-derived neurotrophic factor signalling [7].

Observational and interventional syntheses have generally reported small protective associations [8,9], with one meta-analysis noting that the association was clearer in studies without biological flaws [10]; an umbrella meta-analysis integrating both designs reached the same conclusion [11]. The most recent randomised-trial meta-analysis pooled 20 trials and reported a standardised mean difference of −0.36 [12], while a 2025 systematic review characterised supplementation as a promising adjunct in major depressive disorder [13]. Importantly, the benefit in trials appears concentrated among participants who are deficient at baseline, which raises the possibility that the underlying exposure–response relationship is not linear.

Nevertheless, several methodological issues complicate this research. Cross-sectional designs, which predominate, cannot distinguish stable inter-individual differences from within-person change over time. Studies that use repeated measures frequently treat observations as independent, which underestimates standard errors. Sleep disturbance—a highly prevalent and potent determinant of depressive symptoms in working populations—is seldom modelled explicitly, so vitamin D may absorb variance that properly belongs to sleep. Finally, entering 25(OH)D as a linear term imposes a graded dose–response relationship that may not correspond to the underlying physiology.

To address these issues, we analysed a 12-wave occupational health examination cohort of 62,989 Korean adults (246,751 examinations; 2014–2025). Our objectives were to evaluate the independent association between 25(OH)D and CES-D depressive symptoms using cluster-robust hierarchical regression, to characterise the shape of that association using categorical and restricted cubic spline models rather than assuming linearity, to quantify the relative contribution of sleep disturbance and behavioural factors, and to decompose CES-D variance into stable between-person and time-varying within-person components.

## 2. Materials and Methods

### 2.1. Study Design and Participants

This retrospective longitudinal cohort study used health examination records from a single occupational health screening centre in the Republic of Korea collected over twelve annual waves (wave 1, 2014; wave 12, 2025). Records were eligible if serum 25(OH)D, CES-D score and PSQI global score were all available, yielding 246,751 examinations from 62,989 individuals. The median number of examinations per individual was 3 (mean 3.9, range 1–17); the number of examinations can exceed the number of waves because 1.1% of person-years contained more than one examination. Participants underwent standardised fasting blood sampling, anthropometry and validated self-report questionnaires at each visit.

**Figure 1:**
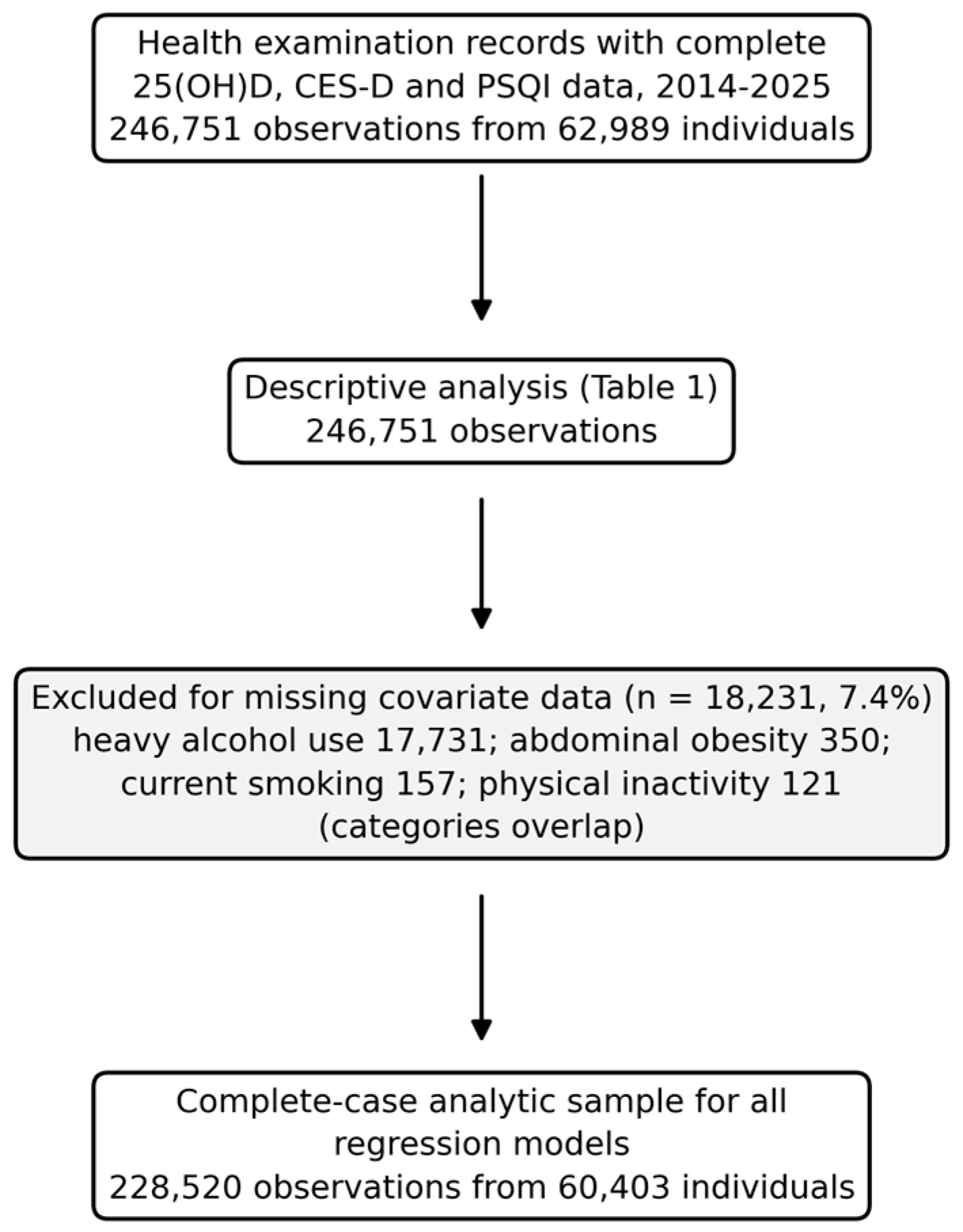
Flow of health examination records through the study.

All regression models used complete-case analysis. Of the 246,751 examinations, 18,231 (7.4%) lacked at least one covariate—principally self-reported heavy alcohol use (*n* = 17,731), abdominal obesity (*n* = 350), current smoking (*n* = 157) and physical inactivity (*n* = 121), with overlap between categories—leaving an analytic sample of 228,520 examinations from 60,403 individuals (Figure 1). Descriptive statistics (Table 1) are presented for the full 246,751 examinations; all regression results refer to the 228,520-observation analytic sample.

The study was conducted in accordance with the Declaration of Helsinki. The protocol was reviewed and approved by the Institutional Review Board of Samsung Changwon Hospital, Sungkyunkwan University School of Medicine (approval number SCMC IRB 2026-06-014; approval date 9 June 2026), which waived the requirement for informed consent because the analysis used de-identified retrospective records.

### 2.2. Exposure: Serum 25-Hydroxyvitamin D

Blood was drawn in the morning after an overnight fast. Samples were kept at room temperature and centrifuged within 1 h (not exceeding 2 h) of collection; serum was then stored at room temperature and assayed within one day. Serum total 25-hydroxyvitamin D (25(OH)D2 + 25(OH)D3) was measured by an electrochemiluminescence binding assay (ECLIA; Roche Diagnostics, Mannheim, Germany), with an inter-assay coefficient of variation below 5%. Over the 12-year study period, the analyte and method principle remained unchanged, while the platform and reagent were updated: examinations from 2014 to April 2016 used the Modular E170 analyser (Vitamin D total reagent). Starting from May 2016, the Cobas 8000 (e602 module) was used, with the Vitamin D total reagent used until to December 2017, the second-generation Elecsys Vitamin D total II from 2018 to August 2022, and the third-generation Elecsys Vitamin D total III from September 2022 onward. Reference-interval verification was performed at each analyser transition and confirmed no change in reference values. Throughout the study, the laboratory complied with the external quality-assessment scheme of the Korean Association of External Quality Assessment Service (KEQAS). Concentrations were categorised as deficiency (<20 ng/mL), insufficiency (20–<30 ng/mL) and sufficiency (≥30 ng/mL) following the Endocrine Society clinical practice guideline [14]. The guideline does not define a separate severe category; we also considered severe deficiency at <10 ng/mL, a cut-off point widely used in the vitamin D literature and corresponding approximately to the fifth percentile of this cohort, in order to test whether risk is concentrated at the extreme of the distribution. 25(OH)D was modelled continuously (per 1 ng/mL), categorically (sufficiency as reference), and as a restricted cubic spline with four knots at the 5th, 35th, 65th and 95th percentiles [15].

### 2.3. Outcome: Depressive Symptoms

Depressive symptoms were assessed with the 20-item Center for Epidemiologic Studies Depression Scale (CES-D; range 0–60), covering depressed affect, somatic complaints, positive affect and interpersonal difficulties over the preceding week [16]. The Korean adaptation has established reliability and validity [17]. The continuous CES-D score was the outcome for linear models. For logistic models, the primary binary outcome was CES-D ≥ 16, the conventional international cut-off point for clinically significant depressive symptoms [16]. This threshold is intended for screening rather than diagnosis and is deliberately sensitive, so scores below it indicate few or only transient symptoms, whereas the higher ≥21 threshold is a more conservative screening cut-off point that favours specificity. Because validation work in Korean samples has suggested higher optimal cut-off points—20/21 for screening and 24/25 for correspondence with clinical diagnosis, reflecting culturally different responses to positive-affect items [18]—all logistic analyses were repeated using CES-D ≥ 21 as a pre-specified sensitivity analysis.

### 2.4. Sleep Quality

Sleep quality was assessed with the Pittsburgh Sleep Quality Index (PSQI), which comprises 19 self-rated items generating seven component scores summed to a global score of 0–21 [19]. A global score ≥ 6 was classified as sleep disturbance for descriptive purposes; the continuous global score was used in all regression models.

### 2.5. Covariates

Covariates were specified a priori: age (years); sex; abdominal obesity (waist circumference ≥ 90 cm in men and ≥85 cm in women); current smoking; heavy alcohol use (defined, following the Korean National Health Screening Program high-risk drinking criterion, as ≥7 standard drinks in men or ≥5 in women on a typical drinking occasion on ≥2 days per week); physical inactivity (failure to meet aerobic activity guidelines); clinician-diagnosed hypertension and diabetes mellitus; season of examination (spring, March–May; summer, June–August; autumn, September–November; winter, December–February, as reference); and survey wave (waves 2–12 versus wave 1) as fixed-effect indicators capturing secular trends including the COVID-19 pandemic period.

### 2.6. Statistical Analysis

Descriptive statistics were stratified by 25(OH)D category and compared using one-way analysis of variance and chi-squared tests. Four hierarchical ordinary least-squares models were fitted sequentially: Model 1, 25(OH)D alone; Model 2, plus age and sex; Model 3, plus lifestyle factors; and Model 4, plus PSQI score, comorbidities and season. All models included wave indicators and cluster-robust (sandwich) standard errors clustered on individual identifier, which yield valid inference under arbitrary within-person correlation without imposing parametric random-effects assumptions [20]. Logistic models with the same covariate structure and the same clustering estimated odds ratios.

Departure from linearity was tested by a likelihood-ratio test comparing the spline model with the linear model. Effect modification by sex was assessed with a fully interacted model in which every covariate, not only 25(OH)D, was allowed to interact with sex; constraining the remaining covariates to a common effect produces a spurious interaction term when covariate effects themselves differ by sex, as they do in this cohort. Sex-stratified models were fitted for presentation. The intraclass correlation coefficient was estimated from an unconditional random-intercept linear mixed model (restricted maximum likelihood) fitted to the full analytic sample. Within-person and between-person correlations between 25(OH)D and CES-D were computed after person-mean centring among individuals with at least two examinations. Significance was set at α = 0.05 (two-sided). All statistical analyses were performed using SPSS version 26.0 (IBM Corp., Armonk, NY, USA) and verified using Python 3.12 (statsmodels v0.14). Robustness of the primary threshold association to the unbalanced repeated-measures structure was examined in pre-specified sensitivity analyses: a population-averaged generalised estimating equation with exchangeable working correlation, a model restricted to one observation per individual, and a model weighting each observation by the inverse of the individual’s number of examinations (Table S1).

## 3. Results

### 3.1. Participant Characteristics

The cohort comprised 246,751 examinations from 62,989 individuals (mean age 43.5 ± 8.2 years; 64.9% of examinations were of men). Mean 25(OH)D was 21.08 ± 8.71 ng/mL: 5.4% of examinations fell below 10 ng/mL and 51.3% below 20 ng/mL. CES-D ≥ 16 was recorded in 10.5% of examinations and CES-D ≥ 21 in 5.9%; sleep disturbance (PSQI ≥ 6) was present in 30.2%.

Table 1 shows characteristics by 25(OH)D category. The severe deficiency group had the highest mean CES-D (7.90 ± 8.34) and the highest crude prevalence of CES-D ≥ 16 (14.1% versus 10.0% in the sufficiency group). This group differed markedly from the others in composition: only 40.9% of its examinations were performed on men, compared with 63.2–70.9% elsewhere, and its mean age was seven years lower than that of the sufficiency group. Because both female sex and younger age are associated with higher CES-D scores in this cohort, the crude contrast is strongly confounded, and the adjusted analyses below offer an appropriate basis for inference.

### 3.2. Hierarchical Linear Regression

Table 2 presents the hierarchical sequence (*n* = 228,520). Crude—each 1 ng/mL increment in 25(OH)D was associated with a 0.027-point lower CES-D score (95% CI −0.033 to −0.020). Adjustment for age and sex halved the estimate (−0.014), and the fully adjusted coefficient was −0.012 (95% CI −0.017 to −0.006; adjusted R^2^ = 0.167). In practical terms, the difference between a person at 10 ng/mL and a person at 30 ng/mL corresponds to about 0.23 CES-D points on a 60-point scale.

PSQI score dominated the model (β = 1.071 per point, 95% CI 1.045–1.097), an association roughly 90 times larger per unit than that of 25(OH)D. Male sex (β = −0.920), current smoking (0.404), physical inactivity (0.380), heavy alcohol use (0.260) and abdominal obesity (0.225) were each independently associated with CES-D score, whereas hypertension and diabetes were not. Relative to winter, spring was associated with a higher CES-D score (β = 0.271, 95% CI 0.147–0.394) and autumn with a slightly lower score (−0.158), while summer showed no difference.

### 3.3. Logistic Regression for CES-D ≥ 16

In the fully adjusted logistic model (*n* = 228,520; prevalence 10.5%; Nagelkerke R^2^ = 0.187), each 1 ng/mL increment in 25(OH)D was associated with an odds ratio of 0.996 (95% CI 0.993–0.999, *p* = 0.011). The PSQI score again dominated (OR 1.372 per point, 95% CI 1.360–1.383), corresponding to a 37% increase in odds per point of the sleep index. Physical inactivity (OR 1.178), current smoking (1.155), abdominal obesity (1.119) and heavy alcohol use (1.094) were independent risk factors; male sex (0.697) and older age (0.975 per year) were associated with lower odds. Relative to winter, spring carried higher odds of depression (OR 1.099, 95% CI 1.033–1.168); summer and autumn did not differ significantly (Table 3).

### 3.4. Shape of the Exposure–Response Relationship

Modelling 25(OH)D categorically showed that the association was not graded (Table 4). Relative to sufficiency (≥30 ng/mL), severe deficiency was associated with a 0.50-point higher CES-D score (95% CI 0.28–0.71) and with 1.20-fold odds of CES-D ≥ 16 (95% CI 1.09–1.32, *p* < 0.001). Deficiency (OR 1.05, 95% CI 0.98–1.12) and insufficiency (OR 1.02, 95% CI 0.96–1.08) were not significantly associated with depression, although the trend across ordered categories was significant (*p* = 0.007).

**Figure 2:**
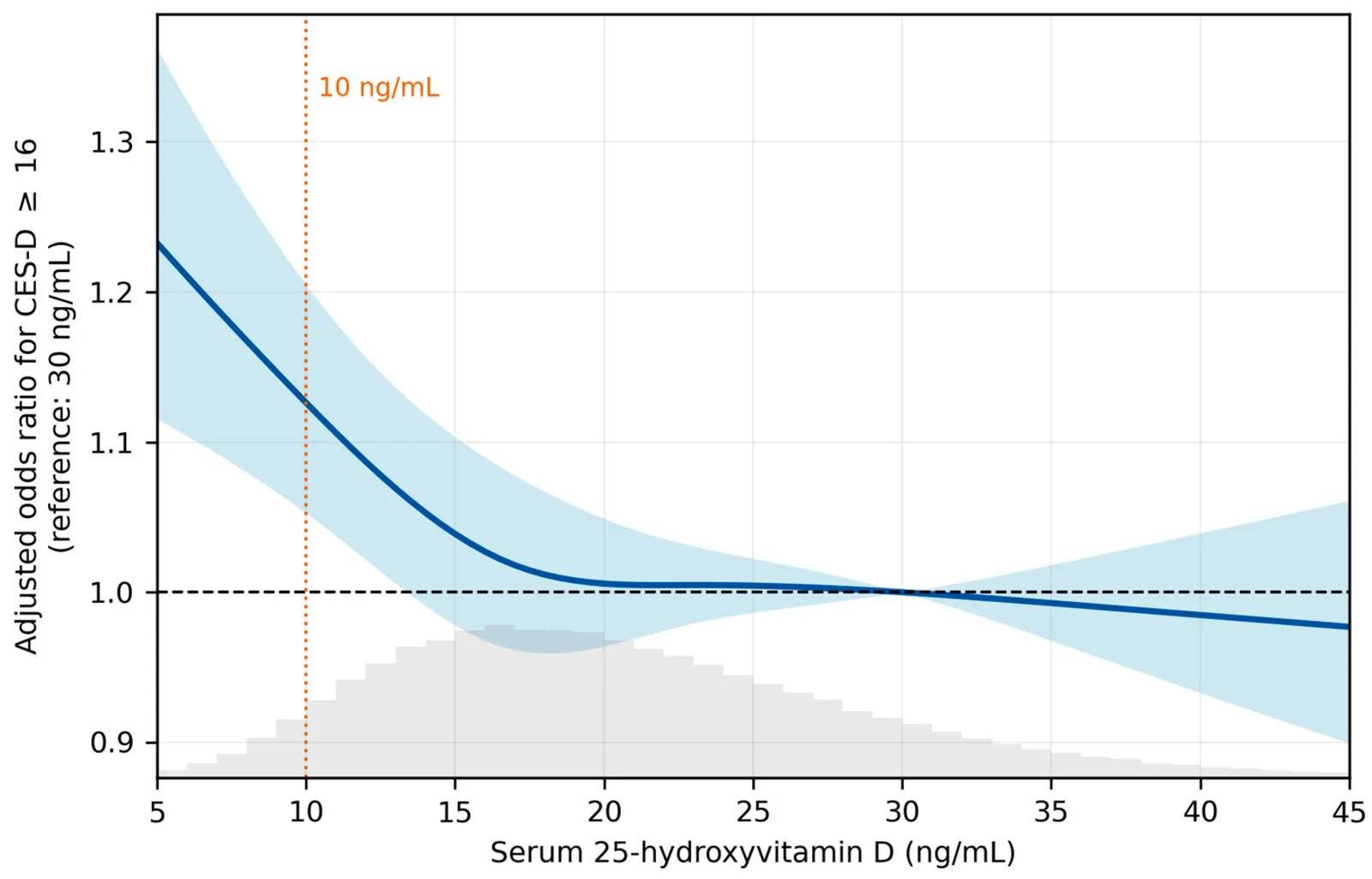
Adjusted odds ratio for clinically significant depressive symptoms (CES-D ≥ 16) across serum 25-hydroxyvitamin D, modelled as a restricted cubic spline with four knots (9.8, 16.8, 22.9 and 36.4 ng/mL) and referenced to 30 ng/mL. The shaded band is the 95% confidence interval from cluster-robust standard errors; the grey histogram shows the distribution of 25(OH)D. Excess risk is confined to the lower tail of the distribution.

The restricted cubic spline model confirmed departure from linearity (likelihood-ratio χ^2^ = 14.1 on 2 degrees of freedom, *p* = 0.001). Taking 30 ng/mL as reference, the adjusted odds ratio was 1.23 (95% CI 1.12–1.36) at 5 ng/mL and 1.13 (95% CI 1.05–1.21) at 10 ng/mL but was indistinguishable from unity from approximately 15 ng/mL upwards (1.04 at 15 ng/mL, 1.01 at 20 ng/mL) and essentially flat across the remainder of the range (Figure 2). Excess risk was therefore concentrated below roughly 12–15 ng/mL.

### 3.5. Effect Modification by Sex

In the fully interacted model, in which every covariate was permitted to differ by sex, the 25(OH)D × sex interaction was not significant for CES-D score (β = 0.004, 95% CI −0.008 to 0.015, *p* = 0.53) or for CES-D ≥ 16 (ratio of odds ratios 1.002, *p* = 0.50). Stratified estimates were correspondingly similar in men (β = −0.010, 95% CI −0.017 to −0.002) and women (β = −0.008, 95% CI −0.017 to 0.002), with the wider confidence interval in women reflecting the smaller number of observations rather than a different effect (Table 5). We note that several other covariate effects did differ substantially by sex—for example, age was inversely associated with CES-D score in women but not in men—which is why an interaction model restricting those covariates to a common effect can generate a misleading 25(OH)D × sex term.

For the severe deficiency category, the adjusted odds ratio was 1.17 (95% CI 1.03–1.34) in women and 1.03 (95% CI 0.89–1.20) in men. Given the non-significant formal interaction, this difference should be regarded as hypothesis-generating.

### 3.6. Variance Decomposition

The unconditional random-intercept model gave a between-person variance of 42.3 and a within-person variance of 14.6, an intraclass correlation coefficient of 0.743. Among the 38,388 individuals with at least two examinations (206,505 observations), the between-person correlation between individual mean 25(OH)D and individual mean CES-D was −0.046 (*p* < 0.001), while the within-person correlation between deviations from personal means was −0.008 (*p* < 0.001)—approximately one-sixth as large. Nearly three quarters of the variance in depressive symptoms is therefore attributable to stable characteristics of individuals, and the component of the vitamin D association that could plausibly respond to supplementation is the smaller within-person component.

### 3.7. Sensitivity Analysis Using the Korean Cut-Off Point

Repeating the logistic analyses with CES-D ≥ 21 (prevalence 5.9%) gave materially unchanged results: the continuous association was OR 0.996 per ng/mL (95% CI 0.992–1.000, *p* = 0.035), and severe deficiency remained associated with depression (OR 1.19, 95% CI 1.05–1.34, *p* = 0.006), while deficiency (OR 1.02) and insufficiency (OR 0.98) did not (Table 6). The threshold pattern is therefore not an artefact of the cut-off point chosen.

### 3.8. Sensitivity to the Repeated-Measures Structure

Across all sensitivity analyses the severe-deficiency association was materially unchanged (Table S1). The population-averaged generalized estimating equation gave an adjusted odds ratio of 1.13 (95% CI 1.06–1.21); restricting the analysis to a single observation per individual gave 1.13 (0.99–1.29); and weighting each individual to an equal total weight, irrespective of the number of examinations contributed, gave 1.25 (1.12–1.39). The continuous per-1 ng/mL association was likewise stable (odds ratios 0.995–0.997 across methods). The threshold effect is therefore not an artefact of individuals who underwent more examinations.

## 4. Discussion

In this analysis of 246,751 repeated examinations from 62,989 working adults, serum 25(OH)D was inversely associated with depressive symptoms, and the association was non-linear. Elevated risk was localised to the severely deficient minority (<10 ng/mL; adjusted odds ratio 1.20), whereas the deficient and insufficient ranges, which together comprise more than half of the study population, showed no independent association. These findings are inconsistent with a graded dose–response and indicate that clinical attention should be directed primarily toward severe deficiency.

The identification of a threshold may help account for inconsistencies in the literature. Supplementation trials tend to show benefit chiefly in cohorts with low baseline 25(OH)D [12,21], whereas large trials and Mendelian randomisation studies conducted in largely replete populations frequently report null findings [22,23]. Our spline model is consistent with these observations: if risk rises only below approximately 12–15 ng/mL, then trials and genetic analyses conducted mainly within the sufficient or moderately insufficient range would be expected to be null, as observed.

Despite statistical significance in a sample of this size, the absolute magnitude of the vitamin D association is modest. In the linear model the difference between 10 and 30 ng/mL corresponds to approximately 0.23 CES-D points, which has limited clinical relevance in isolation; the significance reflects the number of observations (228,520) rather than a substantial effect. Even the categorical estimate, an odds ratio of 1.20 confined to about 5% of examinations, corresponds to a small attributable fraction at the population level. Vitamin D is therefore better regarded as a marker to be corrected in the severely deficient than as a population-level antidepressant strategy.

Sleep quality was a considerably more prominent determinant. The effect of a one-point increase in the PSQI (OR 1.372 per point) substantially exceeded that of 25(OH)D in every model, consistent with meta-analytic evidence that insomnia prospectively predicts incident depression [24]. Although vitamin D and sleep may share serotonergic and circadian pathways [25], the majority of the modifiable variance in this population was attributable to sleep. Occupational mental health programmes may therefore achieve greater effect by prioritising sleep-directed interventions, including cognitive behavioural therapy for insomnia, over population-wide micronutrient supplementation.

The seasonal pattern did not conform to a simple winter-deficiency model. Relative to winter, examinations conducted in spring were associated with higher CES-D scores (OR 1.099) after adjustment for 25(OH)D, whereas summer and autumn did not differ. Spring elevations in depressive symptoms have been described in East Asian populations and attributed to photoperiodic change rather than to circulating vitamin D [26]. Because 25(OH)D itself varies seasonally, the persistence of this association after adjustment is consistent with pathways independent of vitamin D synthesis.

The variance decomposition provides additional methodological context. The high intraclass correlation (0.743) indicates that depressive symptom scores are governed largely by stable inter-individual characteristics rather than state-dependent fluctuation. The within-person correlation between 25(OH)D and CES-D (r = −0.008) was approximately one-sixth of the between-person correlation (r = −0.046), implying that longitudinal change in an individual’s vitamin D concentration corresponds to negligible change in depressive symptoms. Because supplementation acts on the within-person pathway, cross-sectional associations overstate its potential effect, and fixed-effects panel designs or trials restricted to severely deficient participants are the appropriate next step.

The behavioural covariates were consistent with prior evidence. Physical inactivity (OR 1.178) accords with meta-analytic evidence that physical activity reduces incident depression [27], and heavy alcohol use (OR 1.094) with prospective evidence linking alcohol use disorder to later depressive symptoms [28]; both are consistent with the lifestyle-clustering framework for depression [29]. The lower odds observed with older age are common in employed cohorts and probably reflect a healthy-worker survivor effect rather than a protective effect of ageing.

Our threshold finding contrasts with two large cohorts that modelled 25(OH)D differently. In the UK Biobank, a study of middle-aged adults of similar age to our cohort reported that both deficiency and insufficiency were prospectively associated with incident depression (adjusted odds ratios of 1.30 and 1.11, respectively) [30], and a dose–response meta-analysis of prospective cohorts in older adults found an approximately linear inverse association (hazard ratio of 0.88 per 10 ng/mL, with no evidence of non-linearity) [31]. Our data diverge from these graded patterns, with excess risk confined to severe deficiency. Several differences may contribute: the UK Biobank analysis did not adjust for sleep quality, which was the dominant predictor in our models and is itself correlated with vitamin D status; our cohort is younger and occupationally active, with a comparatively low prevalence of the most severe deficiency; and differences in latitude, assay, and outcome instrument may alter the apparent shape of the association. These comparisons reinforce the value of modelling non-linearity explicitly rather than assuming a graded dose–response.

The direction of the association also warrants a comment, since our design cannot establish it and both pathways are biologically plausible: low 25(OH)D might contribute to depressive symptoms, or depressive symptoms might lower 25(OH)D through reduced outdoor activity and sunlight exposure. Three features of our data bear on this question. First, the within-person correlation between change in 25(OH)D and change in CES-D was very small (r = −0.008): when an individual’s vitamin D concentration changed over time, depressive symptoms changed negligibly. Because within-person change is also attenuated by measurement variability in a single-timepoint biomarker, this pattern is at least as consistent with reverse causation, shared confounding, or regression dilution as with a causal effect. Second, the confinement of any excess risk to severe deficiency, together with null Mendelian-randomisation and supplementation findings across the normal-to-replete range, argues against a meaningful causal effect above approximately 15 ng/mL. Third, the modest excess risk below 10 ng/mL could reflect a genuine effect in that range, reverse causation (strongest among the most withdrawn and inactive individuals, who are also the most severely deficient), or residual confounding, and our observational design cannot distinguish these. Disentangling direction will require designs that fix the temporal order—randomised supplementation restricted to severely deficient individuals, within-person fixed-effects or cross-lagged panel analyses, and Mendelian randomisation focused on the low range—and we therefore regard the threshold finding as hypothesis-generating for such targeted studies rather than as evidence of causation.

Several limitations should be acknowledged. The observational design precludes causal inference, and reverse causation is particularly relevant for this exposure, since individuals with depressive symptoms may spend less time outdoors and are less physically active, which lowers cutaneous vitamin D synthesis; the concentration of risk in the severely deficient group and the small within-person correlation are both compatible with this mechanism and cannot be distinguished from a causal effect of vitamin D. Residual confounding by dietary intake, supplement use, outdoor occupation, or vitamin D pathway genotype cannot be excluded, and supplement use in particular could bias the severe-deficiency contrast if treated individuals were reclassified. Depressive symptoms were assessed by self-reporting rather than a structured interview, allowing misclassification, although the concordance of the CES-D ≥ 16 and ≥21 analyses is reassuring. Complete-case analysis excluded 7.4% of examinations, predominantly for missing alcohol data, and some bias may remain if these data were not missing at random. The cohort was drawn from a single occupational screening centre, which limits generalisability to non-working, elderly, and clinically depressed populations. Finally, cluster-robust standard errors correct inference for repeated measures but do not separate within-person from between-person effects as fixed-effects models would, a limitation only partly addressed by the variance decomposition.

## 5. Conclusions

This 12-year cohort study of 62,989 Korean workers with 246,751 repeated examinations demonstrates that the association between serum 25-hydroxyvitamin D and depressive symptoms is non-linear, manifesting principally as a threshold effect confined to severe deficiency (<10 ng/mL; adjusted odds ratio 1.20 for CES-D ≥ 16), with no independent association across the deficient and insufficient ranges that include half of this population. The overall clinical magnitude is modest and is substantially exceeded by that of sleep disturbance (OR 1.372 per PSQI point), and the variance in depressive symptoms is driven largely by stable between-person differences, with the within-person vitamin D signal approximately one-sixth of the between-person signal. These results support targeted screening and repletion for individuals with severe vitamin D deficiency rather than population-wide supplementation, and they underscore the primary role of sleep-directed interventions in occupational mental health management.

## Figures and Tables

**Table 1:** Characteristics of 246,751 health examinations by serum 25(OH)D category. Continuous variables are mean ± standard deviation; categorical variables are *n* (%). Percentages for behavioural variables are calculated among examinations with non-missing data. *p*-values are from one-way analyses of variance (continuous) or chi-squared tests (categorical) across the four categories. CES-D, Center for Epidemiologic Studies Depression Scale; PSQI, Pittsburgh Sleep Quality Index.

Characteristic	Severe Deficiency < 10 ng/mL (*n* = 13,237)	Deficiency 10–<20 ng/mL (*n* = 113,346)	Insufficiency 20–<30 ng/mL (*n* = 86,501)	Sufficiency ≥ 30 ng/mL (*n* = 33,667)	*p*
25(OH)D, ng/mL	8.23 ± 1.47	15.44 ± 2.73	24.25 ± 2.79	36.97 ± 7.73	<0.001
CES-D score	7.90 ± 8.34	7.00 ± 7.54	6.72 ± 7.25	6.73 ± 7.39	<0.001
PSQI score	4.65 ± 2.87	4.52 ± 2.74	4.54 ± 2.73	4.71 ± 2.87	<0.001
Age, years	39.6 ± 8.1	42.3 ± 7.9	44.5 ± 7.9	46.7 ± 8.4	<0.001
Body mass index, kg/m^2^	23.56 ± 3.99	24.29 ± 3.63	24.30 ± 3.37	23.57 ± 3.23	<0.001
Waist circumference, cm	83.25 ± 11.00	85.90 ± 10.21	85.93 ± 9.56	83.84 ± 9.13	<0.001
Gamma-glutamyl transferase, U/L	28.92 ± 56.13	35.46 ± 47.72	37.08 ± 47.85	34.65 ± 45.70	<0.001
Male sex	5418 (40.9)	72,143 (63.6)	61,341 (70.9)	21,273 (63.2)	<0.001
CES-D ≥ 16	1868 (14.1)	12,248 (10.8)	8508 (9.8)	3355 (10.0)	<0.001
PSQI ≥ 6	4163 (31.4)	33,699 (29.7)	25,999 (30.1)	10,682 (31.7)	<0.001
Current smoking	2705 (20.5)	31,056 (27.4)	24,807 (28.7)	8241 (24.5)	<0.001
Heavy alcohol use	2364 (19.1)	23,019 (21.8)	18,317 (22.8)	6808 (22.3)	<0.001
Physical inactivity	5771 (43.6)	51,453 (45.4)	37,737 (43.7)	14,691 (43.7)	<0.001
Abdominal obesity	3897 (29.5)	39,435 (34.8)	28,602 (33.1)	8736 (26.0)	<0.001
Metabolic syndrome	1682 (12.7)	16,592 (14.7)	11,894 (13.8)	3511 (10.4)	<0.001
Hypertension	967 (7.3)	12,601 (11.1)	11,213 (13.0)	5000 (14.9)	<0.001
Diabetes mellitus	340 (2.6)	4413 (3.9)	4110 (4.8)	1890 (5.6)	<0.001

**Table 2:** Hierarchical linear regression predicting CES-D score, with cluster-robust standard errors clustered on individual identifier. * *p* < 0.05; *** *p* < 0.001. All models include survey-wave fixed effects. β, unstandardised regression coefficient; CI, confidence interval.

Variable	Model 1 β (95% CI)	Model 2 β (95% CI)	Model 3 β (95% CI)	Model 4 β (95% CI)
25(OH)D, per 1 ng/mL	−0.027 (−0.033, −0.020) ***	−0.014 (−0.020, −0.008) ***	−0.013 (−0.019, −0.006) ***	−0.012 (−0.017, −0.006) ***
Age, per year	—	−0.034 (−0.042, −0.027) ***	−0.026 (−0.033, −0.019) ***	−0.019 (−0.026, −0.013) ***
Male sex	—	−1.191 (−1.338, −1.044) ***	−1.595 (−1.753, −1.437) ***	−0.920 (−1.062, −0.777) ***
Physical inactivity	—	—	0.537 (0.426, 0.649) ***	0.380 (0.279, 0.481) ***
Current smoking	—	—	0.764 (0.612, 0.915) ***	0.404 (0.267, 0.541) ***
Heavy alcohol use	—	—	0.880 (0.754, 1.005) ***	0.260 (0.147, 0.373) ***
Abdominal obesity	—	—	0.334 (0.205, 0.464) ***	0.225 (0.109, 0.342) ***
PSQI score, per point	—	—	—	1.071 (1.045, 1.097) ***
Hypertension	—	—	—	0.150 (−0.023, 0.323)
Diabetes mellitus	—	—	—	0.207 (−0.088, 0.501)
Spring (vs. winter)	—	—	—	0.271 (0.147, 0.394) ***
Summer (vs. winter)	—	—	—	−0.099 (−0.231, 0.033)
Autumn (vs. winter)	—	—	—	−0.158 (−0.285, −0.032) *
Adjusted R^2^	0.001	0.009	0.015	0.167
Observations	228,520	228,520	228,520	228,520

**Table 3:** Fully adjusted logistic regression for clinically significant depressive symptoms (CES-D ≥ 16), *n* = 228,520, with cluster-robust standard errors. The model includes survey wave fixed effects. CI, confidence interval.

Predictor	Odds Ratio	95% CI	*p*
25(OH)D, per 1 ng/mL	0.996	0.993–0.999	0.011
Age, per year	0.975	0.972–0.978	<0.001
Male sex	0.697	0.652–0.745	<0.001
Physical inactivity	1.178	1.121–1.238	<0.001
Current smoking	1.155	1.078–1.238	<0.001
Heavy alcohol use	1.094	1.038–1.152	<0.001
Abdominal obesity	1.119	1.060–1.183	<0.001
PSQI score, per point	1.372	1.360–1.383	<0.001
Hypertension	1.091	0.998–1.193	0.056
Diabetes mellitus	1.021	0.886–1.178	0.772
Spring (vs. winter)	1.099	1.033–1.168	0.003
Summer (vs. winter)	0.983	0.919–1.050	0.604
Autumn (vs. winter)	0.942	0.883–1.006	0.076
Nagelkerke R^2^	0.187	—	—

**Table 4:** Serum 25(OH)D modelled categorically in the analytic sample (*n* = 228,520), adjusted for age, sex, physical inactivity, current smoking, heavy alcohol use, abdominal obesity, PSQI score, hypertension, diabetes mellitus, season and survey wave, with cluster-robust standard errors. * *p* < 0.05; *** *p* < 0.001. *p* for linear trend across ordered categories = 0.007. β, unstandardised coefficient for CES-D score; OR, odds ratio for CES-D ≥ 16; CI, confidence interval.

25(OH)D Category	*n*	CES-D ≥ 16, *n* (%)	Adjusted β (95% CI)	Adjusted OR (95% CI)
Severe deficiency (<10 ng/mL)	12,269	1729 (14.1)	0.496 (0.281, 0.711) ***	1.200 (1.091, 1.320) ***
Deficiency (10–<20 ng/mL)	105,491	11,288 (10.7)	0.163 (0.025, 0.302) *	1.047 (0.976, 1.123)
Insufficiency (20–<30 ng/mL)	80,299	7789 (9.7)	0.085 (−0.034, 0.205)	1.018 (0.956, 1.084)
Sufficiency (≥30 ng/mL)	30,461	3015 (9.9)	Reference	Reference

**Table 5:** Sex-stratified association between 25(OH)D and depressive symptoms, adjusted for age, physical inactivity, current smoking, heavy alcohol use, abdominal obesity, PSQI score, hypertension, diabetes mellitus, season and survey wave, with cluster-robust standard errors. *p*-values are given for the linear (β) and logistic (OR) models respectively. The interaction row is from a model in which all covariates were permitted to interact with sex. CI, confidence interval; OR, odds ratio.

Subgroup	Observations (Individuals)	β per 1 ng/mL (95% CI)	OR per 1 ng/mL (95% CI)	*p*
Men	151,942 (36,239)	−0.010 (−0.017, −0.002)	0.998 (0.993, 1.002)	0.009/0.26
Women	76,578 (24,166)	−0.008 (−0.017, 0.002)	0.998 (0.994, 1.002)	0.13/0.24
Overall	228,520 (60,403)	−0.012 (−0.017, −0.006)	0.996 (0.993, 0.999)	<0.001/0.011
Interaction with sex	—	0.004 (−0.008, 0.015)	1.002	0.53/0.50

**Table 6:** Sensitivity of the principal findings to the depression cut-off point, *n* = 228,520, fully adjusted models with cluster-robust standard errors. CI, confidence interval; OR, odds ratio.

Outcome Definition	Prevalence	25(OH)D per 1 ng/mL, OR (95% CI)	Severe Deficiency vs. Sufficiency, OR (95% CI)
CES-D ≥ 16 (primary)	10.5%	0.996 (0.993–0.999)	1.200 (1.091–1.320)
CES-D ≥ 21 (Korean screening cut-off point)	5.9%	0.996 (0.992–1.000)	1.186 (1.050–1.340)

## Data Availability

The data supporting the findings of this study are not publicly available owing to privacy and institutional data-governance restrictions. Requests for access may be directed to the corresponding author (chchae@naver.com).

## Supplementary Materials

The following supporting information can be downloaded at: https://www.mdpi.com/article/10.3390/nu18183012/s1. Table S1: Sensitivity of the vitamin D–depression association to the unbalanced repeated-measures structure. All models are fully adjusted for age, sex, current smoking, heavy alcohol use, physical inactivity, abdominal obesity, Pittsburgh Sleep Quality Index score, hypertension, diabetes mellitus, season, and survey wave.

## Abbreviations

**25(OH)D,** 25-hydroxyvitamin D; CES-D, Center for Epidemiologic Studies Depression Scale; CI, confidence interval; ICC, intraclass correlation coefficient; OR, odds ratio; PSQI, Pittsburgh Sleep Quality Index; REML, restricted maximum likelihood; SD, standard deviation.
