# Supplementary material for: Serum 25-Hydroxyvitamin D and Depressive Symptoms in a 12-Year Korean Occupational Cohort: A Threshold Effect Confined to Severe Deficiency
Source: Nutrients. 2026 Sep 15;18(18):3012. doi: 10.3390/nu18183012 (PMC13610108; doi:10.3390/nu18183012)
Supplement: Supplementary file 1 [file nutrients-18-03012-s001.zip › nutrients-4521024-supplementary.pdf]

Supplementary Materials

Serum 25-Hydroxyvitamin D and Depressive Symptoms in a 12-Year Korean Occupational Cohort: A Threshold Effect Confined to Severe Deficiency

Si-Ho Kim, Seung-Hwan Kim and Chang-Ho Chae

Table S1. *Sensitivity of the vitamin D–depression association to the unbalanced repeated-measures structure. All models are fully adjusted for age, sex, current smoking, heavy alcohol use, physical inactivity, abdominal obesity, Pittsburgh Sleep Quality Index score, hypertension, diabetes mellitus, season, and survey wave.*

Analysis	Severe deficiency (<10 vs ≥ 30 ng/mL), OR (95% CI)	Per 1 ng/mL, OR (95% CI)
Primary — pooled logistic regression with cluster-robust standard errors (n = 228,520)	1.20 (1.09–1.32)	0.996 (0.993–0.999)
Generalized estimating equation, exchangeable working correlation (population-averaged)	1.13 (1.06–1.21)	0.997 (0.995–0.999)
One observation per individual (first examination; n = 60,403)	1.13 (0.99–1.29)	0.996 (0.993–1.000)
Examination-count weighting (each individual weighted to a total of 1)	1.25 (1.12–1.39)	0.995 (0.993–0.998)
Person-level analysis using individual mean values (n = 60,403)	—	0.990 (0.986–0.994)

OR, odds ratio for a Center for Epidemiologic Studies Depression Scale score ≥ 16; CI, confidence interval. The analytic sample comprised 228,520 examinations from 60,403 individuals (mean 3.78 examinations per person; median 2; range 1–17), of whom 36.4% (22,015) contributed a single examination. The primary model is the cluster-robust logistic regression reported in the main text. The generalized estimating equation and the examination-count weighting were pre-specified sensitivity analyses; the examination-count weighting (highlighted) assigns every individual an equal total weight regardless of the number of examinations contributed, and corresponds to the approach suggested during peer review. The severe-deficiency association was materially unchanged across all methods (odds ratio 1.13–1.25), and the continuous association was stable (odds ratio 0.995–0.997 across examination-level models; 0.990 in the person-level analysis). The threshold effect is therefore not an artefact of individuals who underwent more examinations.
